# Neurocircuitry of Predatory Hunting

**DOI:** 10.1007/s12264-022-01018-1

**Published:** 2023-01-27

**Authors:** Zheng-Dong Zhao, Li Zhang, Xinkuan Xiang, Daesoo Kim, Haohong Li, Peng Cao, Wei L. Shen

**Affiliations:** 1 School of Life Science and Technology, ShanghaiTech University, Shanghai, 201210 China; 2 Boston Children’s Hospital, Harvard Medical School, Boston, MA 02115 USA; 3 National Institute of Biological Sciences (NIBS), Beijing, 102206 China; 4 Britton Chance Center for Biomedical Photonics, Wuhan National Laboratory for Optoelectronics, Huazhong University of Science and Technology, Wuhan, 430074 China; 5 MoE Key Laboratory for Biomedical Photonics, Collaborative Innovation Center for Biomedical Engineering, School of Engineering Sciences, Huazhong University of Science and Technology, Wuhan, 430074 China; 6 Department of Cognitive Brain Science, Korea Advanced Institute of Science & Technology, Daejeon, 34141 South Korea; 7 MOE Frontier Research Center of Brain & Brain-machine Integration, School of Brain Science and Brain Medicine, Zhejiang University, Hangzhou, 310058 China; 8 Affiliated Mental Health Centre and Hangzhou Seventh People`s Hospital, Zhejiang University School of Medicine, Hangzhou, 310013 China

**Keywords:** Predatory hunting, Neurocircuits, Sensory processing, Sensorimotor transformation, Appetitive motivation, Sequential encoding

## Abstract

Predatory hunting is an important type of innate behavior evolutionarily conserved across the animal kingdom. It is typically composed of a set of sequential actions, including prey search, pursuit, attack, and consumption. This behavior is subject to control by the nervous system. Early studies used toads as a model to probe the neuroethology of hunting, which led to the proposal of a sensory-triggered release mechanism for hunting actions. More recent studies have used genetically-trackable zebrafish and rodents and have made breakthrough discoveries in the neuroethology and neurocircuits underlying this behavior. Here, we review the sophisticated neurocircuitry involved in hunting and summarize the detailed mechanism for the circuitry to encode various aspects of hunting neuroethology, including sensory processing, sensorimotor transformation, motivation, and sequential encoding of hunting actions. We also discuss the overlapping brain circuits for hunting and feeding and point out the limitations of current studies. We propose that hunting is an ideal behavioral paradigm in which to study the neuroethology of motivated behaviors, which may shed new light on epidemic disorders, including binge-eating, obesity, and obsessive-compulsive disorders.

## Introduction

The struggle for survival is the major driving force of the evolution of species, as proposed by Darwin in his book *On the Origin of Species*. Animals cannot survive without food, and carnivorous animals acquire food through predatory hunting, making predatory hunting one of the diverse forms of the struggle for survival. However, natural food availability is often inconstant, and nutrient deficiency is commonplace, so this was a driving force for animals to evolve robust and rigid neural networks to integrate internal signals and external food cues to control hunting motivation. Therefore, predatory hunting is a suitable paradigm for studying sensory processing, and the initiation, and execution of appetitive behaviors. Neuroethological studies of predatory hunting have greatly expanded owing to the advances in new tools for circuitry analysis over the past two decades, revealing the underlying circuit mechanisms of predatory hunting in both teleosts (e.g., larval zebrafish) and rodents (e.g., mice and rats). Larval zebrafish are widely used to decode the neural basis of hunting behavior [1–4]. Meanwhile, mice have emerged as a model in which to reveal the neuroethology of hunting in mammals [5–10]. This review summarizes recent key neurocircuitry discoveries underlying different aspects of hunting neuroethology and emphasizes that predatory hunting is a behavioral paradigm for studying appetitive motivation.

## Ethological Analyses of Predatory Hunting

The behavioral patterns of predatory hunting have been experimentally studied in teleost, frog, bird, and rodents. Predatory hunting involves a series of sequential stereotypical actions, including sensory detection of prey and orienting to prey, as well as approaching/chasing, attacking, biting, and consuming the prey. These behavioral events can be conceptualized into two phases: the goal (prey)-directed appetitive phase and the goal-achieved consummatory phase [11].

**Fig. 1:**
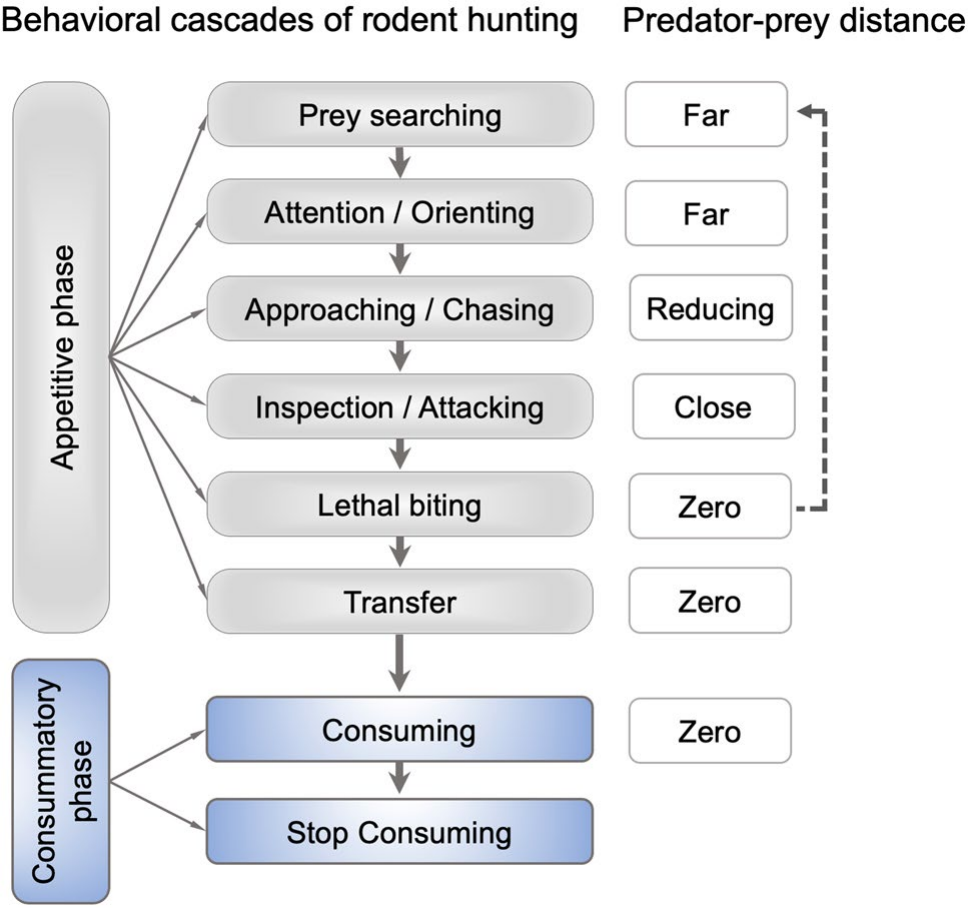
Behavioral ethogram of rodent hunting. Appetitive behaviors are composed of actively orienting, chasing, attacking, and lethal biting and are followed by consummatory behaviors. The predator-prey distances are indicated on the right.

Taking rodents to hunt small insects as an example, as illustrated in Fig. 1, hunting initiates with the sensory detection of the prey before encountering it, followed by a latency during which the predator orients itself toward the prey. After this, the predator actively pursues the prey, beginning with prey-oriented movement and ending with close contact with the prey. Once the prey and predator are engaged to the point where the prey-predator distance is nearly zero, the predator begins to vigorously attack the prey using its mouth or forepaws until the prey is subdued. Before being subjugated by the predator, the prey may also aggressively fight with the predator and attempt to escape. As a result, the above actions may occur several times until the prey is captured by the predator or successfully evades capture. Finally, successful hunting terminates with the prey being consumed or retained for future consumption [5, 8, 9, 12]. Predatory hunting is regarded as an innate behavioral response to prey. Meanwhile, experience significantly increases hunting efficiency. Therefore, hunting is a composite of innate and learned components [13]. Although rodent hunting actions manifest substantially consistent patterns, distinctive behavioral adaptations have also been reported among different species. For instance, different species of *Cricetinae* (hamsters) have been shown to employ distinctive manipulation strategies, such as seizing, handling, and nibbling [14].

**Fig. 2:**
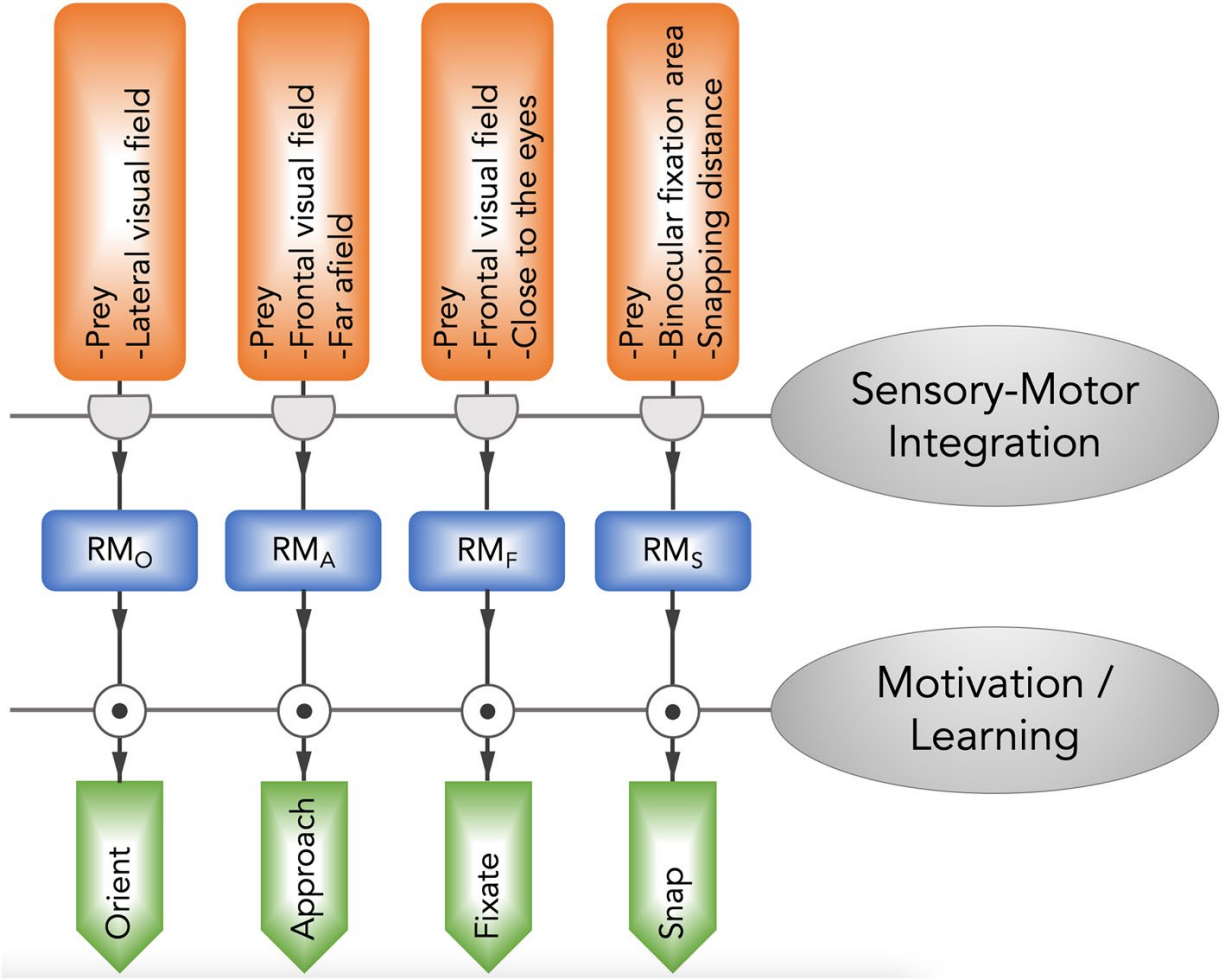
Action patterns of toad hunting behavior. The sensory input of prey and its location are processed by the appropriate releasing mechanisms (RMs) and lead to various hunting-related actions: orienting (O), approaching (A), fixating (F), and snapping (S). The stimulus-release-action sequence is mediated by sensory-motor integration and is modified by motivational status and prior experience. Adapted from [16].

Thorough analyses have revealed the substantial pattern of behavioral components underlying these instinctive hunting actions. The founders of modern ethology, Nikolaas Tinbergen and Konrad Lorenz described the key components of instinctive behaviors: the sign stimulus (also termed releaser), the innate releasing mechanism (IRM), and the fixed-action pattern (FAP) [15]. The sign stimuli, such as visual and tactile cues, are the essential features of the stimuli that are necessary to initiate hunting actions. The IRM refers to the sensorimotor interface that transforms key stimulus inputs into behavioral outputs. Moreover, the FAP is a "hard-wired" or instinctive behavioral sequence triggered by a cue. For example, egg-rolling behaviors in greylag geese for retrieving eggs that have rolled out of the nest and escape behaviors of chicks in response to a hawk-like bird. Multiple sessions of the stimulus-releasing-action sequences are embraced in complex hunting behaviors. Using the toad as the model, the pioneering neuroethologist Jörg-Peter Ewert investigated in detail the neural mechanisms of visual cues that trigger prey-hunting behavior. The patterns of toad hunting behaviors are illustrated in Fig. 2. Depending on prey sensation and their relative locations, the appropriate releasing mechanisms (RM) are activated, and subsequent hunting actions are executed. Toads adjust their location relative to the prey by orienting, where the prey is located in the lateral visual field, or by approaching, where the prey is located within the frontal visual field. When the prey is close to the toad, fixating/snapping actions may be determined following the perception of prey-predator distance. Once the prey is within the binocular fixation area, prey-catching is achieved by snapping. These action patterns are robust and rigid, and they are significantly influenced by motivation and learning [16]. Similar behavioral cascades are also seen in rodent hunting [12], as shown in Fig. 1. Together, animals incorporate genetically determined instinctive behavioral sequences with external prey cue stimuli, and this incorporation is modified by motivational state, experience gained, and learning. This incorporation enables robust and stereotypical, as well as flexible hunting actions, which are critical for individual survival in a complex environment.

The stimulus-release-action sequence has been described in many species. For example, a moving worm-like black bar is sufficient to elicit prey-capture behavior in toads [17]. In larval zebrafish, small moving spots are capable of triggering hunting-like behaviors, including eye movements and tail turns [18]. However, this hypothesis has not been quantitatively examined in rodent hunting, despite some studies reporting hunting-like behaviors (for instance, interaction, chasing, and attacking) towards prey-like objects [5–7, 9, 19]. It would be worth systematically investigating whether a single sensory modality is capable of triggering hunting behavior in rodents.

**Fig. 3:**
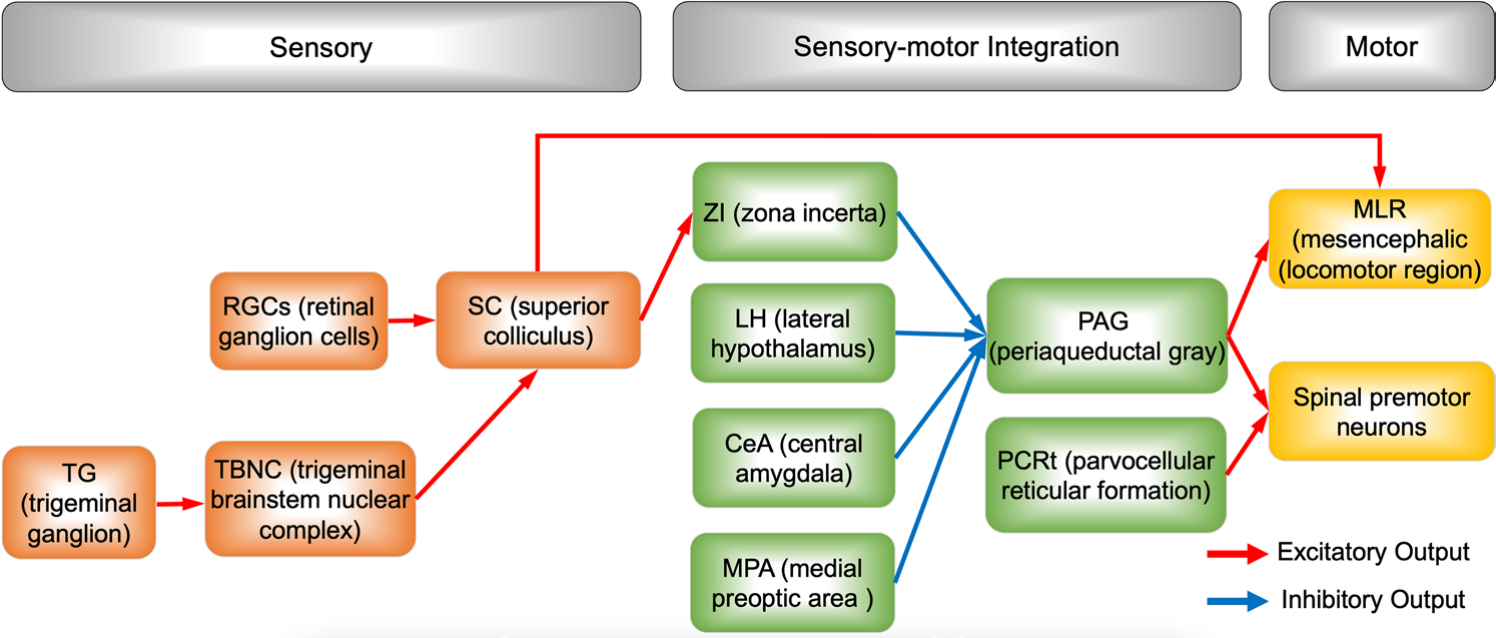
Summary of the neurocircuits underlying predatory hunting. The regions are involved in sensory processing, sensory-motor integration, and motor execution. The red and blue arrows indicate excitatory and inhibitory neuronal connections, respectively.

**Fig. 4:**
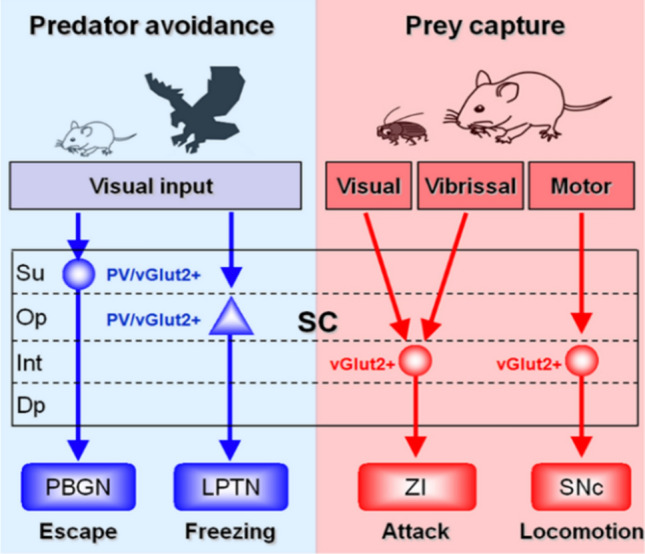
Summary of the “Yin-Yang” circuit modules in the mouse SC for the survival behaviors of predator avoidance and prey capture. Blue indicates “Yin” and red indicates “Yang” circuit modules. Single-cell RNA sequencing shows the distinct gene expression in SC neuronal subtypes. For detailed information, see [94]. Su, superficial grey layer of the SC, Op, optic nerve layer of the SC, Int, intermediate gray layers of the SC, Dp, deep gray layer of the SC.

**Fig. 5:**
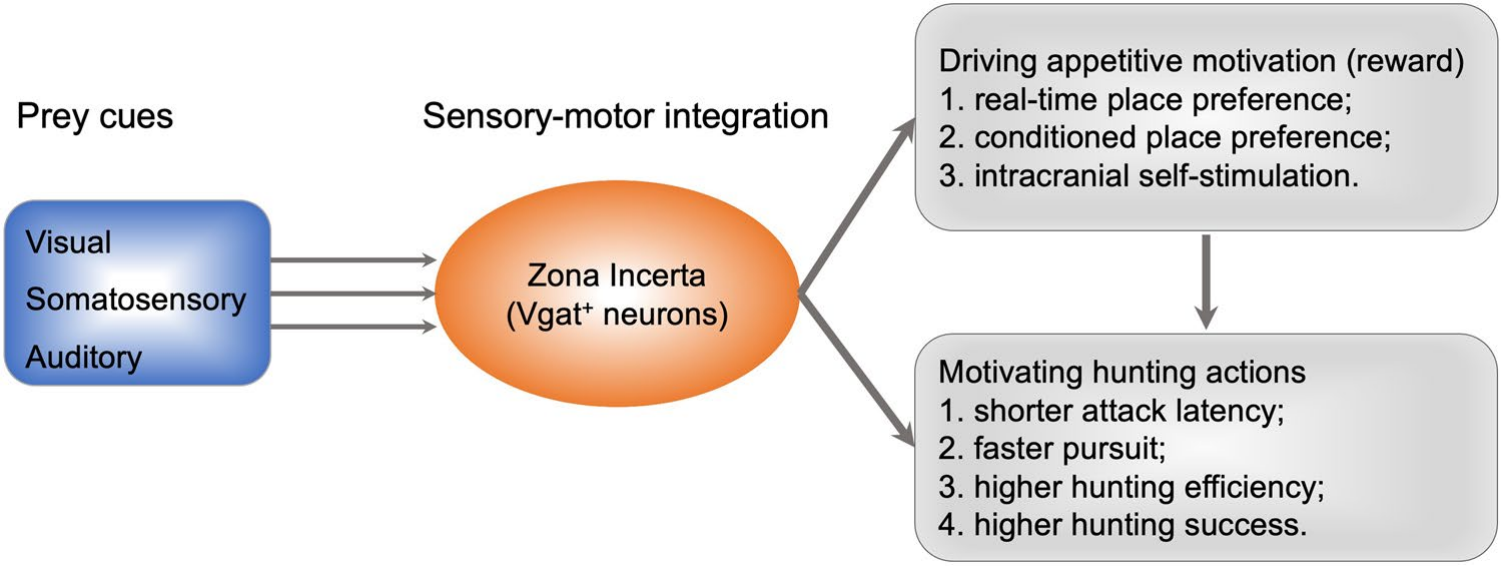
The zona incerta integrates prey-related sensory signals and promotes hunting through an appetitive motivation mechanism. GABAergic ZI neurons are activated by multiple prey-associated cues, which are intrinsically rewarding and further energize hunting behaviors.

Despite considerable similarities between the teleost and rodent hunting behaviors described above, they also show several differences, including the motivation for predation and sequential motor encoding (sensory processing of prey-related features, sensorimotor transformation in predation, motivation for predation, and sequential encoding of hunting actions) [18, 20]. First, more sensory modalities may be involved in rodent hunting compared with larval zebrafish; for instance, whisker-mediated tactile sensory signals [21]. Second, mammalian species display more complicated motor patterns in predatory hunting, such as orienting, attacking, grabbing, and eating, which forms a motor sequence lasting at least 1–2 minutes. Third, mammalian species need to overcome the prey defense and kill the prey, which is driven by the motivation for predation. Fourth, sequential encoding of hunting efficiently organizes intrinsic actions of hunting and makes the process more flexible and robust. Many brain nuclei have been identified to encode these aspects of hunting, including the superior colliculus (SC), the central nucleus of the amygdala (CeA), the lateral hypothalamus (LH), the zona incerta (ZI), the medial preoptic area (MPOA), the periaqueductal gray (PAG), the mesencephalic locomotor region (MLR), the reticular formation, and spinal premotor neurons. These nuclei form sophisticated neurocircuits to achieve delicate control of hunting behavior (Fig. 3). The following text focuses on recent advances in the neuroethology and neural circuits of predatory hunting in mice and compares some of these results with those of zebrafish larvae (Figs. 4 and 5).

## Sensory Processing of Prey-Related Features

Studies of neuroethological mechanisms begin with identifying key sensory modalities that are involved in predatory hunting. Depending on different habitats, diurnal/nocturnal lifestyles, and physical profiles, predatory hunting is carried out under the guidance of various sensory modalities in vertebrates. Among these sensory modalities, one or more specific modalities dominate hunting in most cases, while other sensory modalities may be recruited by predators for compensation when the primary sensory modality is dysfunctional [22]. In mammalian models, the participation of sensory cues in predatory hunting, including prey-derived visual, vibrissal tactile, auditory, and olfactory cues, has been investigated in shrews [21, 23, 24], moles [25], mice [20, 26–28], and rats [29]. Yet, how auditory and olfactory cues are involved in hunting has not been investigated comprehensively in the mouse model. In the following paragraphs, we elaborate on how sensory cues are detected by sensory organs and processed in the downstream sensory pathways in the brain.

### Visual Pathway

The visual pathway has been shown to be important in hunting [8, 20, 30]. For example, retinal ganglion cells (RGCs) that are sensitive to object motion, detect visual features of prey [31] including size, location, motion, and contrast polarity [8, 30]. Interestingly, a visual “releasing signal” can trigger the predatory motor sequence in toads [17], where a visual bar that moves in a direction parallel to the toad's body orientation is sufficient to elicit a predatory attack [17]. Consistent with that reported in toads, local-edge detector RGCs in rabbits detect targets with a contrasting border when it appears or moves in the receptive-field center [32, 33]. It has been reported that mouse W3 RGCs are similar to local-edge-detectors in rabbits [34], showing a preference for moving targets and acting as a type of “bug detector” [35]. However, the evidence is inadequate to illuminate their role in predatory hunting. Both zebrafish and mouse track prey with a binocular visual field [18, 36]. Mouse hunting benefits from binocular vision through stereopsis, facilitating depth perception and accurate estimation of prey locations. Binocular vision also improves the resolution of mouse vision and enhances sensitivity under dim light and low contrast [36–38]. A subset of ipsilaterally-projecting RGCs and SC neurons have been implicated in processing binocular inputs and guiding predation [36, 39].

RGCs innervate diverse downstream visual centers [40, 41], including the SC, which can be divided into multiple layers with different functions in sensory processing and sensorimotor transformation [42]. The superficial layers of the SC receive inputs from the retina and these connections are topographical, with the dorsoventral axis of the retina projecting to the lateromedial axis of the SC, and the temporonasal axis of the retina projecting to the rostrocaudal axis of the SC [43, 44]. SC neurons have specific visual response properties, and most SC neurons prefer flashing spots or drifting gratings 10–20° in diameter [45, 46]. Several studies have revealed that diverse subtypes of SC neurons in the superficial layers show distinct properties in response to visual stimuli [47, 48]. For example, the wide-field (WF) neurons respond selectively to small moving objects through a large region of space [47, 48], and are suitable for detecting prey. The narrow-field (NF) neurons with small spatial receptive fields are direction-selective [47], making them ideal for reporting the precise location of moving prey. Indeed, WF and NF neurons were reported to participate in predatory hunting in mice in a recent study. The WF neurons are responsible for prey detection and initiation of approach, while the NF neurons are involved in accurate orienting during pursuit [10].

### Vibrissal Tactile Pathway

Predatory hunting in several mammalian species is also associated with vibrissal tactile cues detected by the whiskers [21, 23–25, 29]. The movement of prey results in the deflection of whiskers and generates tactile cues, thus activating neurons responding to mechanical stimuli in the trigeminal ganglions [49]. The trigeminal ganglia then send tactile signals to the trigeminal complex in the brainstem [50], consisting of four subnuclei: Sp5c, Sp5i, Sp5O, and Pr5. Sp5i, Sp5O, and Pr5 send projections to neurons in the deep layer of the SC, forming clustered projection fields [51, 52], and transmitting tactile signals sensed by the whiskers to neurons in the SC [53]. Therefore, it is worth exploring whether the Sp5/Pr5–SC pathway relays the somatosensory signals to the SC to guide predatory hunting, considering that the SC plays a key role in predatory hunting [8, 10, 29, 54].

### Multisensory Integration

Predatory hunting in several mammalian species relies on multiple sensory modalities, as reviewed above. The precise approach to the prey requires both visual and auditory inputs [20]. The efficiency of predatory hunting can be further reduced when simultaneously trimming the whiskers and leaving the mice in darkness compared to that when depriving them of only visual or vibrissal somatosensory inputs [8, 9], suggesting that predatory hunting benefits from the integration of different sensory modalities. Multisensory integration can be achieved at the single neuron level [55, 56]. However, studying how the brain integrates multisensory cues to optimize hunting efficiency is just beginning. For example, some neurons in deep layers of the SC respond to multiple sensory stimuli [57, 58]. Notably, some glutamatergic SC neurons in the deep layers that project to the ZI receive visual and vibrissal somatosensory inputs simultaneously and promote hunting in mice [8], suggesting that these neurons play a role in detecting prey motion. Like the SC, ZI neurons also integrate visual and vibrissal somatosensory signals and promote hunting [9]. However, besides the two types of the sensory signal, the ZI also integrates auditory signals, which has not been reported in the SC neurons projecting to the ZI [8]. These visual and vibrissal somatosensory signals are incorporated into prey detection and hunting [9]. These results suggest that more hunting-related sensory modalities are integrated as they move down the SC–ZI pathway, which would help the discrimination of prey and increase hunting efficiency.

### Comparison with Zebrafish

We wondered whether prey detection mechanisms would be conserved among vertebrates by comparing studies on zebrafish and rodents. First, in zebrafish, RGCs that respond to prey stimuli project to both the OT and pretectum. Yet, these pretectum-projecting RGCs have not been reported to respond to prey stimuli in rodents, so this is worthy of further investigation. Second, the lateral-line system in zebrafish can sense the water flow generated by zooplankton and hunt them even in the dark [59–61]. Some neurons in the zebrafish tectum respond to non-visual stimuli, such as water flow, and this may be the underlying circuit mechanism for hunting zooplankton in the dark [62]. Thus, although there are many differences between mice and zebrafish in prey-detection circuits, the processing (or integrating) of visual and tactile cues during hunting is a shared mechanism.

## Sensorimotor Transformation in Predation

Predatory hunting consists of a chain of motor actions starting with prey-related sensory detection and often ending with a lethal attack. After prey detection, the predator orients the eyes, head, and body toward the prey in coordination and then starts to chase and attack the prey [20, 63]. The process in which the brain converts sensory information into motor actions is known as sensorimotor transformation. It is critical for animals to execute appropriate goal-directed actions during prey chasing, attacking, and biting. In addition, outcomes from the motor system can be used to adjust the sensory response to future stimuli. This flexible sensorimotor integration enables animals to hunt efficiently in complex environments. For example, in zebrafish, prey evokes convergent eye movements to accurately target the prey [18, 20]. It remains unclear whether mice use the same strategy as zebrafish [18, 64]. Recordings of the eye movements of rodents during predatory hunting by using a miniaturized head-mounted camera may help to find the answers [65]. In the following section, we discuss the neuronal substrates that mediate the processes of sensorimotor transformation.

### Superior Colliculus

The SC is a midbrain structure that responds to visual, somatosensory, and auditory cues to guide spatial orienting [66–71]. In earlier studies, rats exhibited contralateral movements when the SC, especially its lateral part, was electrically or pharmacologically stimulated, which is similar to the process of orienting and pursuing a moving object [72–74]. In addition, microinjections of picrotoxin into the SC produce “biting-like” actions, resembling predatory jaw attacks on prey [75]. The SC is associated with active navigation [76]. These findings enlightened investigators to explore the function of the SC in hunting [29, 54].

The SC may encode the spatial signal through maps to control hunting-related motor actions. For example, the SC neurons of mice are distributed in a three-dimensional map in the deep layers, representing spatial head movements [77]. This map is genetically determined, integrates multiple sensory cues, and accurately controls the turning of the head for orienting responses [78]. In addition, a motor map has been proposed for deciphering saccadic eye movements in the mouse SC, although this map has not been fully completed [79]. Similarly, in rodents, SC neurons have been found to participate in controlling locomotion, despite the absence of a definite locomotor map plotted in the SC to trigger predatory hunting and other behaviors [67, 76]. The sensory and motor maps are well aligned in the SC [71], and the potential implications of intra-SC networks in predatory hunting need to be determined in further studies.

SC neurons send projections to many motor-related regions, and this may be a neuronal substrate by which the SC integrates the different motor actions of predatory hunting [66, 80]. First, the contralaterally crossed descending tectofugal pathways may mediate the head movement during orienting, which is a major part of hunting initiation [81]. Second, the descending projections from the SC to the MLR, the lateral paragigantocellular nucleus [82], and the gigantocellular reticular nucleus [83] may control locomotion [84–86]. In support of this hypothesis, preliminary data suggest that activation of the SC–MLR pathway evokes locomotion, and SC neurons projecting to the MLR are activated when the predator starts to approach the prey [8]. Third, the SC neurons also send axons to the parvicellular reticular formation of the medulla oblongata [87], and the ventral part of the medullary reticular formation [88], which are involved in gnawing and skilled forelimb motor tasks, respectively. These pathways correspond to the jaw attack and paw attack during predatory hunting. Further studies are needed to determine whether the SC simultaneously triggers paw attacks and jaw attacks through collateral projections. Fourth, some SC neurons form a tectorial pathway that triggers dopamine release in the dorsal striatum and promotes prey pursuit [89]. It would be of great importance for future studies to determine how the above-described tectofugal pathways collectively encode the sequential motor actions during predatory hunting.

Investigation of the interaction of circuits for prey capture and predator avoidance is essential. SC neurons project to many regions, including the lateral posterior thalamic nucleus [90, 91], the parabigeminal nucleus [92], and the ventral tegmental area [93], forming several pathways to evoke freezing and escape in repose to a visual stimulus. Studies have suggested that SC neurons that are involved in visual predator-mediated avoidance behavior are genetically different from those that participate in prey-capture behavior [94]. Future studies are required to define the synaptic connections between these two subtypes of SC neurons. In summary, the “Yin-Yang” circuit modules have been proposed to illustrate the distinct pathways mediating predator avoidance and prey capture *via* neurons expressing distinct marker genes in the mouse SC. It would be of great importance to extend studies under various environmental contexts and to determine how molecularly-defined circuits signal visual, tactile, auditory, or other sensory information to guide adaptive avoidance and capture actions.

### Zona Incerta

The ZI was previously proposed to execute sensory-linked ‘global functions’ that involve diverse processes, such as arousal, attention, locomotion, and visceral activity [95]. However, the global functions were not determined. Recently, several lines of evidence support the idea that predatory hunting could be one of these global functions. First, the ZI has intensive connections with sensory processing centers (such as the SC and the thalamus) [96] and motor control centers (such as the periaqueductal gray and cerebellum) [97, 98], which makes it an ideal hub to integrate prey-related sensory signals and subsequently generate neural activity to promote predatory attacks. Second, single-unit recording of a large population of ZI neurons has identified subsets of neurons that are sensitive to multiple sensory signals of prey, and half of the neurons are also activated by movement [9]. These co-excited neurons might be good candidates for sensorimotor transformation. Moreover, direct stimulation of ZI neurons strongly promotes jaw attack action [9]. Altogether, the ZI is a promising site for sensorimotor transformation to control hunting actions, and further studies need to focus on decoding how ZI neurons orchestrate the sensory and motor components.

### Periaqueductal Gray

The PAG is essential for survival behaviors, including predatory hunting and defensive behavior [99–101]. On one hand, the PAG is involved in the sensory processing of these behaviors. During hunting, neurons of the lateral columns of the PAG (LPAG) are recruited for sensory detection and target discrimination [99]. These neurons also participate in risk assessment [102]. On the other hand, the PAG controls various motor components of survival behaviors. The circuit from the CeA to the PAG controls locomotion during pursuit [5]. Recently, a study found that neurons in the LPAG are divided into seven clusters with differing dynamics during hunting [99]. The activity of LPAG neuronal ensembles is time-interlaced for the entire behavioral process and forms a sequential chain of activity to encode the hunting motor program. These findings suggest that PAG modulates multiple complex motor actions related to fear, anxiety, and reward-seeking *via* different neuronal populations [100, 103, 104]. Also, the firing of LPAG neurons is time-locked to the activity of jaw muscles during biting [105]. Thus, as a functional interface of survival behaviors between limbic structures and the lower brainstem and spinal cord, the PAG integrates sensory information and converts signals into distinct motor outputs [101, 106]. The role of the PAG is considered to coordinate behavior in preparation for prey or danger. However, the detailed mechanism of the stimulus-response loop of hunting needs further exploration with a multi-dimension high-speed motion capture system and animal postural analysis [107].

### Cortex and Basal Ganglia

In most cases, the prey does not stand still during predatory hunting, making the predator pursue and attack the fleeing prey requires the prediction of the future position of the prey to increase the efficiency of hunting. Many species of both invertebrates [108] and vertebrates have the ability to track the position of prey, including amphibia [109], archerfish [110], zebrafish [111], and non-human primates [112]). Visual cues may be the substrate for neural computations, from the retina [113] to the cingulate cortex [112]. Moreover, the cortical–striatal–thalamic circuit is associated with motor planning, learning, and execution [114]. Specifically, lesions of the ventrolateral striatum reduce feeding efficiency, and lesions of the dorsolateral striatum impair forelimb motor control [115]. Inactivation of the ventrolateral part of the dorsal striatum in rats impairs predatory attacks with both paws and jaw, thus reducing hunting efficiency [116, 117]. A recent study found that cell-type-specific pathways from basal ganglia to the MLR control locomotion [84], while whether these pathways regulate the pursuit of prey remains to be determined.

### Motivation for Predation

Wild animals face more challenges than those in laboratory conditions. On one hand, predators need lots of energy and make efforts to overcome the prey's defenses and kill it. For example, cheetahs have to run at a top speed of 93 km/h to catch a running impala [118]. On the other hand, as a classic idiom says: “the mantis stalks the cicada, unaware of the oriole behind” predators are also exposed to danger during hunting and could potentially be targeted by higher-order predators. Together, the hunting process is high-risk, requires lots of energy, and must be driven by strong motivation [119]. Earlier researchers identified motivation-related brain areas in mammals by using c-Fos mapping [120, 121]. In recent studies, diverse functions of these areas in predatory hunting have been revealed by modern circuit analysis tools. Notably, specific subsets of neurons localized in these brain areas, e.g., the CeA, LH, and ZI, have also been implicated in food consumption (CeA [122]; LH [123]; ZI [124]; PAG [125]).

### Central Nucleus of the Amygdala

The CeA is a hub to encode defensive and appetitive behaviors [126]. In the CeA, specific subsets of neurons control the consumption of food [122, 127, 128] and fluid [129]. Strikingly, CeA neurons regulate premotor neurons that control the movement of the jaws, tongue, and laryngopharynx in an indirect manner [130], providing a possible neural basis for the regulation of predatory hunting in rodents. Indeed, optogenetic and chemogenetic activation of CeA neurons induces predatory-like attacks on both insect and artificial prey [5]. Strikingly, the CeA neurons projecting to the PAG encode approaching, and the CeA neurons that send projections to the reticular formation in the brainstem, are involved in the predatory attack [5]. The putative role of the CeA in predatory hunting needs to be investigated in detail. First, do the CeA neurons that promote food consumption and predatory hunting belong to the same subtype? Second, how does the CeA–PAG pathway trigger the pursuit of prey [5] and defensive behavior [100]?

### Lateral Hypothalamus

The LH is considered to be a feeding center that controls food consumption in both rodents [123, 131] and zebrafish [132]). Notably, a recent study has elucidated the potential role of GABAergic LH neurons in predatory attacks in mice [6]. These neurons are activated during hunting, and their optogenetic activation drives predatory attacks when prey is implicated. In addition, a recent study revealed that GABAergic LH neurons promote predation to some extent by suppressing defensive responses [133]. Interestingly, the hypothalamus may also function as a feeding center in predatory hunting in zebrafish [134]. Real-time imaging revealed the activation of neurons in the hypothalamic feeding center when zebrafish are exposed to prey-like visual stimuli. Synaptic inactivation of these hypothalamic neurons by neurotoxin injection reduces prey consumption. The underlying mechanisms of how the LH mediates feeding and hunting need further investigation.

### Medial Preoptic Area

Besides the LH, other regions in the hypothalamus are involved in predatory hunting. A critical study has demonstrated that mice vigorously engaged with 3D objects and chased moving objects when the CaMKIIa neurons in the MPOA projecting to the vPAG are activated [7]. Interestingly, action induced by the MPOA–vPAG circuit does not occur unless the target appears within the binocular visual field, highlighting the critical role of prey sensation in initiating hunting. This study deciphered the generation of the motivation to acquire a 3D object during predatory hunting. Moreover, recent studies have revealed that a vGluT2^+^ excitatory MPOA–vPAG circuit and a GAD65^+^ inhibitory MPOA–vPAG circuit modulate exploratory behavior in opposite directions [135, 136]. These studies showed that the MPOA mediates complicated and versatile functions for searching and hunting for targets. In addition, experiments in the MPOA have shown that hunting does not end with consumption. Mice simply kill crickets without consuming them when the MPOA-vPAG circuit is activated, which suggests that this circuit is not linked with hunger-driven hunting, but rather it may be an extension of investigatory behavior. Likewise, future research on hunting circuits can discriminate between these types of motivation by determining whether the hunting evoked by a neural circuit ends with the final consumption of prey (rather than only killing) and whether this behavior is specific for prey or can also be evoked by non-prey food (e.g., chow pellets).

### Zona Incerta

The ZI is a part of the subthalamus and reacts directly to both visceral and somatic stimuli [95]. Indeed, the hunger signal ghrelin derived from the gut can physiologically activate GABAergic ZI neurons [124]. In addition, photostimulation of these neurons results in binge-like eating, implying the critical role of ZI in the transformation of visceral signals into food-consuming behavior. However, it is still unclear how the ZI processes somatosensory cues from the environment. A recent study identified that GABAergic ZI neurons function in the integration of prey-relevant sensory cues, inducing a strong appetitive motivational drive for prey capture [9]. Notably, photoactivation of GABAergic ZI neurons or the GABAergic ZI–PAG projection increases the number of nose-pokes and results in a preference for the stimulated side, indicating the role of ZI in inducing positive motivation [9]. Moreover, the GABAergic ZI–PAG pathway is not responsible for food consumption during predatory hunting, which is different from the role of the ZI–PVT pathway for high-fat diet feeding [124], suggesting that GABAergic ZI neurons trigger appetite and drive consumption *via* distinct pathways. Interestingly, recent research has shown that ZI neurons are linked to novelty-seeking behavior [137, 138]. Therefore, the ZI might play a general role in encoding motivated investigatory behaviors, and hunting might be one of these investigatory behaviors, especially when considering that the ZI– PAG pathway promotes hunting without inducing feeding.

### Periaqueductal Gray

The PAG has been reported to play a role in "quiet biting" attack behavior [139]. Almost two decades ago, the Canteras group reported that insect predation is accompanied by increased cFos expression in the rostrolateral PAG (rlPAG) [140]. Further studies have shown that the rlPAG is involved in reward-seeking during predatory hunting [103, 141], while there are still questions to be answered: how the motivational signals from different brain areas are integrated into the rlPAG, and how the rlPAG sends axons to downstream regions to control pursuit and predatory attack in hunting. Single-cell RNA sequencing could help to classify the neuronal subtypes in the PAG, which may answer the above questions. In addition, the FosTRAP2 mouse line may help to access neurons activated in the rlPAG associated with hunting [142]. Predatory hunting-related regions, including the CeA, LH, MPOA, and ZI, all project to the PAG [5–7, 9], making the PAG a potential center for information integration. These regions form a network for the motivational control of predatory hunting. More recently, this network has been elucidated by multichannel recording, revealing that neuronal activity in the LPAG follows a sequential pattern, providing a framework for decoding complex instinctive behaviors such as predation [99].

There are still many important questions about the roles of the PAG in regulating survival behaviors to be answered. One is how the sensory signals are transmitted to activate motivation for predatory hunting. We may find a clue in the SC–ZI pathway, which serves as a neural substrate in transmitting sensory signals to predatory attacks in mice [8, 9]. Similar to that in mice, a neural pathway from the pretectal area to the lateral hypothalamic zone in zebrafish also participates in the conversion of visual food detection into feeding motivation [134]. The other is how PAG orchestrates complex inputs to guide behaviors for survival. Research has shown that the PAG is closely linked to defensive behaviors, such as flight and freezing [143, 144], so the global function of the PAG may be more linked to controlling the assessment of threats, and the hunting evoked by neural circuits linked to the PAG may in part be linked to scaling down an animal’s perception of threat.

### Sequential Encoding of Hunting Actions

Sequential encoding was first introduced when explaining the neural mechanisms of navigation and motor generation in the prefrontal cortex [145–147], hippocampus [148], and motor cortex [149]. Different from persistent neuronal activity patterns, sequentially organized and transiently active neurons reliably show maze or motor trajectories in the neural space on broader timescales [147]. This form of activity is good for maintaining a high dimensional dynamic and heterogeneous activity across complex behavior [146].

Complex animal behaviors often form sequences that are built from simple stereotyped actions and shaped by environmental cues [107]. Accordingly, the hunting process is divided into four phases in order: search, chase, attack, and consume [9, 99], among which mice perform different actions. Thus, neuronal encoding of the motor program of this hunting behavior needs to integrate information from distinct inputs and form heterogeneous neuronal activity across time. The LPAG is a common downstream target in controlling the hunting motor program [5–7, 9]. It is crucial to know how the LPAG processes information from different inputs during hunting behavior. Single-unit recordings in the LPAG have shown that neurons with a rigorous sequence form seven different hunting phase-locking ensembles to encode the hunting motor sequence. Consistent with the concept of hard-wiring of instinctive behaviors, cell type-specific recordings, and phase-specific optogenetic inhibition have revealed that neuronal ensembles in the motor sequence could be genetically defined [99].

**Fig. 6:**
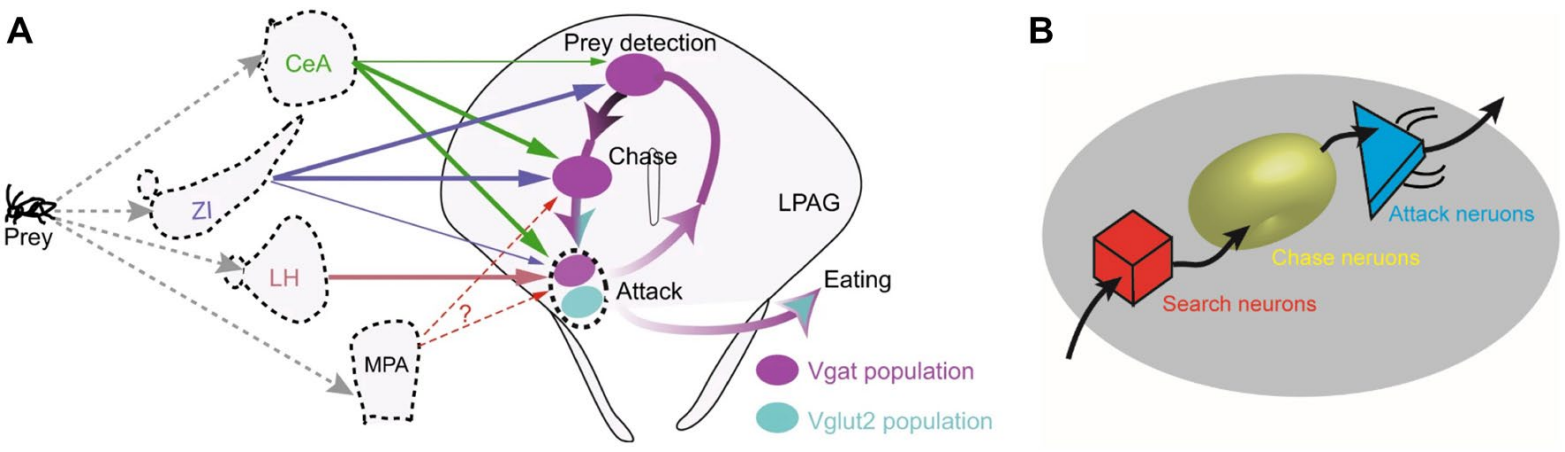
Summary of sequential encoding by the PAG in predator hunting. **A** Distinct PAG neuronal activity patterns form a sequence along with hunting behavior. Hunting-related inputs, such as the CeA, ZI, LH, and MPOA, modulate the behavioral sequence by acting on different PAG neuronal ensembles. **B** Diagram of the sequential encoding model. Predatory hunting motor programs, such as searching, chasing, and attacking, are sequentially encoded in different PAG neuronal ensembles. Adapted from [99].

Unlike the large population of excitatory neurons in the neocortex [150], how LPAG neurons are sequentially recruited in hunting behavior is not clear. As described above, almost all the hunting-related brain nuclei, the ZI, CeA, LH, and MPOA, exert their functions directly *via* the PAG [5–7, 9]. These inputs might play roles in modulating the phase-locking activity sequence. Combining retrograde tracing virus tools and single-unit recordings, researchers specifically ablated GABAergic neurons in the ZI, CeA, and LH projecting to the LPAG, and found these ablations change the activity of PAG hunting ensembles. Ablation of the ZI reduces the activity of all ensembles across hunting, ablation of the CeA mainly reduces the activity of the chase and attack ensembles, and ablation of the LH selectively reduced the activity of the attack ensemble. Thus, LPAG integrates information from different inputs within the sequential framework (Fig. 6). Results from the cross-comparison of behavioral parameters for all three ablations also supported this mechanism. Overall, LPAG neurons are organized into several ensembles to chain actions into a sequence, which we propose to realize both robustness and flexibility. These findings suggest that complex instinctive behaviors are embedded with sequential action sequences [99].

## The Overlap between Hunting Circuitry and Feeding Circuitry

In the laboratory setting, predatory hunting can be considered a special form of feeding behavior. The above-discussed neuronal circuits for hunting have indeed been shown to regulate feeding behaviors. Among them, LH, CeA, and ZI control binge eating [124, 151]. Subtypes of MPOA and PAG neurons regulate feeding behaviors [152, 153]. Notably, the feeding phenotypes are not always consistent among different studies, which might be owing to differences in targeted neurons or feeding measurement assays. For instance, the GABAergic LH projection to the PAG only has mild effects on feeding [6, 152]. Care should be taken regarding the nutrition compositions (e.g., chow, high fat, high sugar, pellet, liquid), the context of food presentation (e.g., home-cage, novel environment, head-fixed), and food intake measurement assays (e.g., time scale, fasting, *ad libitum*) when interpreting the behavioral outcomes of neuronal manipulations. For example, activation of the GABAergic ZI neuronal projection to the PVT induces binge eating of high-fat food rather than high-sugar or chow food [124]. Another interesting finding is that, besides promoting feeding and hunting, stimulation of LH, CeA, and ZI neurons induces gnawing (or fictive feeding) even in the absence of food, suggesting that these neurons also control motor activity. Lastly, optogenetic stimulation of the LH, CeA, and ZI regions generates positive valence and induces self-stimulatory behaviors [9, 122]. Thus, hunting actions without consumption could be driven by appetitive motivation associated with prey or negative motivation associated with hunger.

## Limitations of Current Studies

Predator hunting consists of a series of actions that occur within one minute, and their complexity impedes the decoding of the neuronal mechanisms underlying multiple behavioral variables, such as sensory processing, decision-making, sensory-motor transformation, motor control, and appetitive motivation. The temporal resolution of traditional neuronal manipulation approaches (chemogenetics, drug treatments, and genetic approaches) is inadequate to discriminate these behavioral events. In contrast, the optogenetic approaches provide optimal temporal and spatial resolution. Thus, it has been applied to precisely control the timing and locus of neuronal stimulation during hunting. Besides methodology, it remains unclear whether these behavioral variables are encoded by individual neurons or whether subgroups of neurons are specialized in mediating specific behavioral variables. Well-controlled behavioral paradigms that can discriminate these interconnected behavioral events are needed. Next, current studies have applied various hunting training assays, for example, different insects (cricket or cockroach), and different ages (young or adult), which impede direct comparisons between different studies. It is necessary to standardize and quantify hunting training assays. Finally, current studies mainly focus on a large population of neurons and potentially produce broad behavioral phenotypes. Future studies will combine refined behavioral models with genetic tools, single-cell resolution neuronal recording, and new system neuroscience approaches to advance our understanding of predatory hunting behaviors.

## Summary

Predatory hunting provides an ideal model for studying complex natural behaviors. In this review, we discussed the ethological analysis of predatory hunting behaviors and summarized the neural circuits that mediate behavioral actions of hunting. Visual and vibrissal tactile cues of prey, and possibly auditory cues, are processed by the SC and the ZI to further initiate hunting action cascades. Multiple brain nuclei, including the SC, ZI, CeA, LH, MPOA, and PAG, have been shown to convert different sensory inputs into behavioral outputs and execute pursuing, chasing, attacking, killing, and consuming prey (Table 1). The ZI, MPOA, LH, and PAG regulate the strength and timing of motivational drives that are essential for energizing hunting behaviors. The PAG integrates multiple input signals from the ZI, MPOA, LH, and CeA, and encodes the sequential organization of hunting actions. Furthermore, these nuclei are also required for many individual survival behaviors, such as feeding, social interactions, and defense. Further studies are needed to determine how organisms make decisions and execute behaviors in response to complex rival stimuli by integrating internal psychological states, external stimuli, and experience. Finally, the neural substrates of hunting are still present in modern humans, as the hunter-gatherer lifestyle occupied a long period in the evolution of *Homo sapiens*. Future studies may focus on the dysfunction of hunting circuits and their potential link to eating disorders and obsessive-compulsive disorders.

**Table 1:** Summary of the effects of predatory hunting after optogenetic stimulation of different brain nuclei and their projections

Region	Cell Type	Projection	Results Summary
CeA	Vgat	PCRt	CeA promotes predatory hunting through activating craniofacial musculatures
			Gain/loss of functions studies indicate that CeA→PCRt projections control mandibular and cervical musculatures and mediate prey killing bites
		PAG	Gain/loss of functions studies indicate that CeA→PAG projections control locomotion during prey pursuit
LH	Vgat	PAG	Optogenetic activation of PAG-projecting LH neurons drives predatory hunting
		PAG	Bidirectionally optogenetic modulation of LH GABA neurons regulates predatory attack
	Vglut2		Activating PAG-projecting LH glutamate neurons drives evasion behavior
MPOA	CaMKIIα	PAG	Optogenetic activation of CaMKIIα+ MPA→vPAG circuit promotes object exploration
			Optogenetic activation of CaMKIIα+ MPA→vPAG circuit induces hunting behavior toward natural prey
SC	Vglut2	ZI	Optogentic activation of the SC→ZI pathway provokes predatory hunting
			Single-unit recording reveals that ZI-projecting SC neurons detect prey-derived visual and somatosensory cues
			Fiber photometry recording reveals that ZI-projecting SC neurons transform sensory cues into attack-related neural signals
ZI	Vgat	PAG	Gain/loss of functions studies indicate that ZI GABAergic neurons promote predatory hunting
			Single-unit recording indicates that ZI encodes multiple modalities of prey sensory signals
			ZI GABAergic neurons induce an appetitive motivational drive
			GABAergic ZI→PAG projection is required in hunting
